# Efficacy and safety of Yaobitong capsule for acute lumbar disc herniation: A protocol for a multi-center randomized controlled trial

**DOI:** 10.1097/MD.0000000000031533

**Published:** 2022-11-25

**Authors:** Xianshuai Zhang, Kexin Yang, Siyi Wang, Bin Tang, He Yin, Qunhui Lei, Guohui Zhou, Mingyu Gu, Mingpeng Shi, Changwei Zhao, Shaojun Li, Zhenhua Li

**Affiliations:** a Changchun University of Chinese Medicine, Changchun City, China; b China Academy of Chinese Medical Sciences, Wangjing Hospital, Beijing, China; c Affiliated Hospital of Changchun University of Chinese Medicine, Changchun City, Jilin Province, China.

**Keywords:** durg therapy, lumbar disc herniation, randomized controlled trial, Yaobitong capsule

## Abstract

There are few effective conservative therapies for acute lumbar disc herniation (LDH), and the choice of nonsteroidal anti-inflammatory drugs is not recommended for all patients. The purpose of this study was to compare the effect of Yaobitong capsule with celecoxib capsule, and to further confirm the safety and efficacy of Yaobitong capsule.

**Methods:**

This study is a large sample multicenter randomized controlled trial. Eight hospitals served as sub centers to recruit patients. A total of 258 patients are divided into Yaobitong group and celecoxib group according to the ratio of 1:1. Celecoxib or Yaobitong capsule was taken orally for 14 days. Patients will complete the trial after 3 months of follow-up, and independent statisticians who are blinded to random assignment will analyze the data using SAS 9.3 software. The primary outcome was the visual analogue scale (VAS) score after 14 days of treatment, and Japanese Orthopaedic Association (JOA), Oswestry Disability Index (ODI), and SF-12 will be regarded as secondary outcomes. Safety indexes will be recorded before and after treatment, and adverse events (AEs) will be recorded throughout this trial.

**Discussion:**

This study will evaluate the efficacy and safety of Yaobitong capsule in treating LDH. The experimental results will provide evidence support to treat LDH with Yaobitong capsule.

## 1. Introduction

Lumbar disc herniation (LDH) is a frequently occurring disease in orthopedics, which seriously endangers the physical and mental health of patients.^[1,2]^ Most of the protruding lumbar intervertebral discs compress the nerve roots of L5 and S1, resulting in typical symptoms of sciatica.^[3]^ One study suggests that patients who fail to respond to conservative treatment for 6 weeks meet the surgical indications.^[4]^ At present, although the surgical treatment schemes such as percutaneous endoscopic lumbar discectomy are gradually improved,^[5]^ conservative treatment is still the first choice considering the acceptability of surgical treatment and postoperative complications.^[6–8]^

A large number of meta-analysis and randomized controlled trials showed that acupuncture, massage, traditional Chinese medicine, epidural injections and non steroidal anti-inflammatory drugs have a clear curative effect on LDH, which provides an optional treatment scheme for patients.^[9–13]^ However, a network meta-analysis showed that the effect of oral Chinese medicine is lower than that of acupuncture and massage, but there is insufficient evidence to this conclusion for acute LDH.^[14]^ Recent animal studies have shown that Yaobitong capsule can inhibit the inflammatory response and neuropathy of herniated intervertebral disc.^[10]^ However, there are few clinical studies on the treatment of lumbar process with lumbago, and there is a lack of clinical evidence.

Guidelines of the North American Spine Society and the American College of Physiologists recommend celecoxib as the first-line treatment for acute LDH.^[15]^ Although celecoxib capsules are widely used in the treatment of low back pain, there is evidence that celecoxib does not completely cure these lesions.^[16]^ Moreover, celecoxib lacks sufficient evidence to prove their effectiveness in improving daily function. As we know, celecoxib also has adverse reactions. The most common adverse reactions are gastrointestinal reactions, which increases the risk of gastrointestinal bleeding and perforation.^[17]^ In addition, patients with cardiovascular disease may not be suitable for celecoxib.^[18–20]^ Therefore, this study compared Yaobitong capsule with celecoxib capsule to further confirm the efficacy and safety of Yaobitong capsule, which may provide optional oral drugs for patients with acute LDH.

## 2. Methods and Analysis

### 2.1. Study design

**Figure 1.**
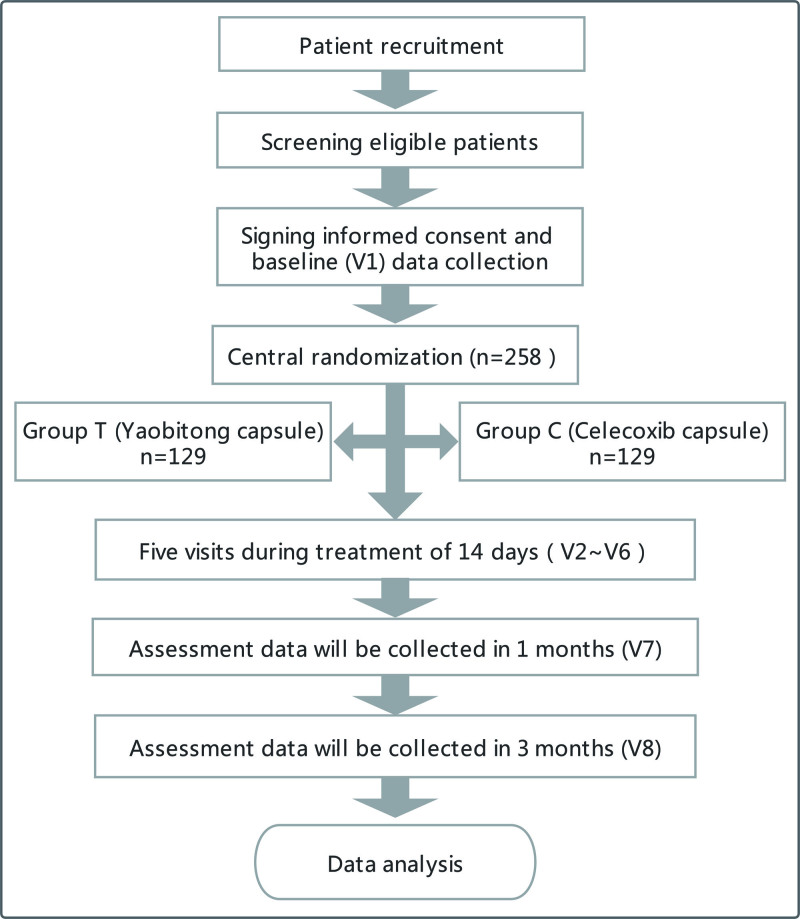
The study flow chart. The flowchart of enrollment, interventions and analysis.

This study is a multi-center, randomized controlled trial with a superiority design. Subjects who met the inclusion conditions will be allocated to the experimental group and the control group in a ratio of 1:1. The experimental group will be treated with Yaobitong capsule and the control group will be treated with Celecoxib capsule. The subjects in the 2 groups have no other basic treatment, and the treatment period is 14 days and the follow-up period was 3 months. The trial process is shown in Figure 1. The study was officially launched on November 8, 2021, and is expected to be completed in February 2023. Eight medical institutions will complete this trial, including The First Affiliated Hospital of Tianjin University of traditional Chinese Medicine, Gansu Hospital of traditional Chinese Medicine, Henan Luoyang orthopedic hospital, Affiliated Hospital of Shanxi University of traditional Chinese Medicine, Dongzhimen Hospital of Beijing University of traditional Chinese Medicine, Shandong Pharmaceutical Biotechnology Research Center, Suzhou Hospital of traditional Chinese Medicine, and The First Affiliated Hospital of Hunan University of traditional Chinese Medicine.

### 2.2. Ethics and registration

This study was approved by the ethics committee of Changchun University of traditional Chinese medicine on June 11, 2021 (Permission number CCZYFYLL2020 Shenzi-016-02), and is valid until June 11, 2023. We will follow the International Conference Guideline for Good Clinical Practice to ensure that the data and the results are credible. Before starting the study, Patients will be informed of the treatment plan and ethical approval number, and they have the right to know the intervention measures of the trial. Informed consent will be signed by the patients. The patients will sign the informed consent (see appendix 1) if they agree to participate in the clinical trial. This study is registered on September 22, 2020 with the Chinese Clinical Trial Registry, and the registration number is ChiCTR2000038405.

### 2.3. Patient recruitment

The study began to recruit patients on November 8, 2021. The first volunteer was recruited on November 29, 2021. It is expected to complete the patient recruitment in November 2022.Eight sub centers from different provinces in China participated in the implementation of the project. The First Affiliated Hospital of Tianjin University of traditional Chinese medicine, Gansu Hospital of traditional Chinese medicine, and Dong zhimen Hospital of Beijing University of traditional Chinese medicine were respectively responsible for the recruitment of 24 patients; Henan Luoyang orthopedic hospital, the Affiliated Hospital of Shanxi University of traditional Chinese Medicine and the First Affiliated Hospital of Hunan University of traditional Chinese Medicine were responsible for recruiting 36 patients respectively; Shandong Medical Biotechnology Research Center was responsible for recruiting 40 patients; Suzhou Hospital of traditional Chinese medicine was responsible for recruiting 38 patients. Each sub center will recruit volunteers through local advertising.

### 2.4. Eligibility criteria

#### 2.4.1. Diagnostic criteria.

The diagnostic criteria of LDH refer to an evidence-based clinical guideline for the diagnosis and treatment of LDH with radiculopathy and The diagnostic efficacy standard of TCM syndrome of the people’s Republic of China.^[9,21]^ The diagnostic criteria are as follows:

1. Medical history: with or without a definite history of lumbar injury.
2. Symptoms and signs: low back pain radiates to buttocks and lower limbs, abdominal pressure increases, such as cough, sneezing, etc; There was tenderness and radiation to the lower limbs, and the lumbar movement was limited; There may be hypersensitivity or dullness in the affected innervated area of the lower limbs. Muscle atrophy may occur in patients with a long course of the disease. Tendon reflex and dorsiflexion of the toe may be weakened.
3. Physical examination: positive muscle strength and sensation test; Straight leg raising or strengthening test was a positive result.
4. Auxiliary examination: MRI showed LDH.

#### 2.4.2. Inclusion criteria.

1. The patient was aged between 18 and 70 years old. The clinical symptoms were leg pain extending below the knee, which was consistent with the corresponding nerve root distribution area of the MRI.
2. The first course of low back pain or leg pain was less than 3 months, or the latest acute recurrence time was less than 3 months.
3. This study will compare the efficacy and safety of Chinese patent medicine and Western medicine in the treatment of LDH.
4. The VAS scores of leg pain were more than or equal to 4.0.

#### 2.4.3. Exclusion criteria.

1. The patient had a history of lumbar surgery.
2. Patients have received oral or epidural steroid therapy in recent 3 months.
3. Severe diabetes with peripheral neuropathy, severe osteoporosis, gastric ulcer, gastrointestinal bleeding, spinal tumor, spinal tuberculosis, spinal compression fracture.
4. Allergic to non-steroidal drugs.
5. There are symptoms of cauda equina nerve damage or parenchymal and progressive loss of motor function.
6. Patients with litigation or legal claims.

### 2.5. Randomization and blinding

This study will apply the central randomization method by using the interactive Web response system to randomly assign the subjects to any group. The personnel providing the random sequence do not participate in the implementation of the whole trial. All clinical trial researchers evaluating outcomes can only get random numbers to assign patients by using computer, and neither the researchers nor the patients know which group the patients will be assigned. The independent statisticians will be blinded to random assignment.

### 2.6. Intervention

The treatment regimen was divided into treatment and controlled groups according to randomization. The treatment group(T) was given oral Yaobitong capsules for 14 days. Usage and dosage: oral administration, 3 capsules each time, 3 times a day after meals. Main functions: promoting blood circulation and removing blood stasis, removing wind and dampness, promoting qi, and relieving pain. Indications: low back pain caused by blood stasis and qi stagnation and closed vein.

The controlled group (C) was given oral Celecoxib capsules for 14 days. Usage and dosage: oral administration, the recommended dosage is 400 mg for the first dose on the first day, which can be increased by 200mg if necessary, and then 200 mg twice a day according to the medication instructions.

### 2.7. Sample size

This study is a randomized controlled trial with parallel control and superiority design. According to previous literature, the VAS score of the control group (celecoxib capsule) after treatment was 5 points.^[22–27]^ It is expected that after 2 weeks of treatment, the difference between the experimental group (Yaobitong capsule) and the control group is 0.5 points, and the standard deviation of the 2 groups is the same (standard deviation = 1.1). It was hypothesis *α* = 0.05 (bilateral), 1-*β* = 0.90. According to the calculation of PASS 11 software, at least 206 cases were collected in this study. Assuming a loss of follow-up rate of 20%, 258 patients need to be included in this study.

### 2.8. Outcome measurements

#### 2.8.1. Primary outcome.

VAS (visual analogue scale) score of leg pain on the 14th day of treatment was the main evaluation index of curative effect. VAS adopts a 10 cm scale divided into 1 to 10 points, of which 10 points are the maximum pain and 0 points are painless.^[28]^ Patients choose the score according to the degree of leg pain, which is the VAS score to evaluate the degree of pain.

#### 2.8.2. Secondary outcome.

Oswestry disability index (ODI) was used to assess dysfunction. The ODI has 10 main items, including 60 sub-items, to evaluate dysfunction from multiple dimensions.^[29]^ The lower the score, the lower the degree of functional restriction, and vice versa.

Japanese Orthopaedic Association (JOA) was used to assess dysfunction and degree of improvement.^[30]^ The JOA total score is 29 and the lowest is 0. The lower the score, the more obvious the dysfunction. The improvement index and improvement rate were used to assess the degree of improvement after treatment.

Improvement index = post-treatment score - pretreatment score.

Improvement rate = [(post-treatment score - pretreatment score)/ 29 - pretreatment score] × 100%.

SF-12 scale is a simplified version of SF-36, which contains 12 items with a full score of 100 to evaluate the physiological and psychological status of patients.^[31]^ The higher the score, the better the patient’s quality of life.

Secondary outcomes included the ODI score, JOA score, and SF-12 score of all visits. In addition, VAS scores at other visit times were also secondary outcomes. All visit points and evaluation indexes are presented in Table 1.

**Table 1:** Visit plan.

Time	0th d(baseline)	1st d	3rd d	5th d	7th d	14th d	1st mo	3rd mo
Visit	V1	V2	V3	V4	V5	V6	V7	V8
**Assessment index**								
Blood test	√					√		
ECG	√					√		
Urine test	√					√		
MRI	√					√		
X-ray	√					√		
JOA	√	√	√	√	√	√	√	√
ODI	√	√	√	√	√	√	√	√
VAS	√	√	√	√	√	√	√	√
SF-12	√	√	√	√	√	√	√	√
Adverse event		√	√	√	√	√	√	√

The “√” represents the implementation.

ECG = electrocardiogram, MRI = magnetic resonance imaging.

#### 2.8.3. Visit plan.

In this study, 8 visit points were designed (see Table 1), including day 0 before treatment (baseline, V1) and 5 visit points during 14 days of treatment (V2 ~ V6). The follow-up time points include month 1 (V7) and month 3 (V8). Each patient will complete all visits on the 3rd month. JOA, ODI, VAS, SF-12 scores will be collected at all interview points. Blood test, urine test, ECG, MRI, and X-ray will be collected at baseline and at the end of the treatment period on day 14. Adverse events (AEs) will be collected at all visit points (V2 ~ V8) during treatment and visit.

### 2.9. Safety assessment

The incidence of AEs was used to assess the safety of the intervention. The AEs of all patients were collected during treatment and follow-up, and the severity of AEs was evaluated. We will report the AEs to the ethics committee, which will decide whether to terminate the trial. The patient’s AEs will be recorded in detail in the case report form for the evaluation of the final results. If the patient has AEs, this study will bear all the medical expenses of the patient to adverse reactions.

### 2.10. Data collection and management

This trial uses a paper-based case report form (CRF) and an electronic data management system. The study staff will collect basic subject information, observational indicators, laboratory indicators, and AEs. Data collators will fill in the above data in the case report form and study medical record.

### 2.11. Statistical analysis

All outcomes were analyzed based on intention-to-treat analysis. The analysis will be carried out with SAS 9.3 software. It is statistically significant that the *P* value of bilateral test is less than 0.05. Multivariate imputation by chained equations method will be used for analysis and data processing of those who violated the trial protocol. Data analysis will be conducted by independent statisticians.

## 3. Discussion

Conservative therapy is the first choice for patients with acute low back pain. Some studies pointed out that physical therapy such as traction, laser, and ultrasound can effectively treat acute LDH, which may provide effective external therapy for patients in the acute stage.^[15,32]^ Although some clinical guidelines highly recommend nonsteroidal anti-inflammatory drugs such as celecoxib and ibuprofen as first-line drugs to low back pain, Yaobitong, ^[9,27]^as a Chinese patent medicine, has been widely used in the clinical treatment of low back pain in China.

Yaobitong capsule contains Panax notoginseng, Ligusticum chuanxiong, Rhizoma Corydalis, Radix Paeoniae Alba, Achyranthes bidentata, Cibotium barometz, cooked Rhubarb and Angelica pubescens.^[33]^ Traditional Chinese medicine believes that Yaobitong capsule has the effects of promoting blood circulation, hemostasis, detumescence, and analgesia, and can effectively treat patients with acute low back pain. Recent studies on the determination of rat plasma components found that the Yaobitong capsule mainly contains ginsenoside Rg1, ginsenoside Rb1, osthol, tetrahydropalmatine, paeoniflorin, paeoniflorin, and ferulic acid, among which the content of ginsenoside from Panax notoginseng is the highest,in recent years, Ginsenoside Rg1 and ginsenoside Rb1 are considered to be effective components for the treatment of LDH.^[34]^ Previously, several randomized controlled trials showed that oral Yaobitong capsule could significantly reduce VAS score, IL-1β and TNF- α in serum, the total effective rate of LDH is more than 95%.^[35–37]^However, the intervention of these trials adopted the scheme of Yaobitong combined with other therapies (e.g., traction, acupuncture). At present, there is deficient evidence of efficacy on the alone application of Yaobitong capsule.

Overall, the current evidence shows that oral Yaobitong capsules have a good effect on LDH, but this conclusion is not confirmed by large sample randomized controlled trials, especially in the treatment of acute LDH. The purpose of this study is to further explore the efficacy and safety of Yaobitong capsules in the treatment of acute LDH and to provide reference evidence for health decision-making. Referring to the guidelines on low back pain, we developed a control group celecoxib, which is recommended as a first-line treatment for acute low back pain. We adopted internationally recognized efficacy measurement methods, including VAS, JOA, ODI, and SF-12 scale. The lumbar dysfunction, degree of pain, and quality of life of all participants will be assessed by these scales. In addition, based on the large sample study of 258 patients, we can explore adverse reactions and safety indexes of Yaobitong capsules to evaluate the safety of Yaobitong in patients with acute LDH.

This study is a large sample multi-center randomized controlled trial, but there are limitations: the blinding is not implemented for participants and outcome assessors in this trial, which may produce potential bias factors. The statisticians who pool all results will be blinded throughout this trial, which may effectively reduce bias. The conclusions of this trial will provide the decision maker with valid information concerning the effect and safety of Yaobitong capsules in patients with acute LDH.

## Author contributions

**Conceptualization:** Zhenhua Li, Xianshuai Zhang, Kexin Yang, Bin Tang, Siyi Wang.

**Data curation:** Xianshuai Zhang, Siyi Wang, Kexin Yang, Changwei Zhao, Shaojun Li, Zhenhua Li.

**Formal analysis:** He Yin, Guohui Zhou, Siyi Wang, Mingyu Gu.

**Investigation:** Xianshuai Zhang, Siyi Wang.

**Methodology:** Zhenhua Li.

**Project administration:** Xianshuai Zhang.

**Software:** Bin Tang, Kexin Yang.

**Supervision:** Zhenhua Li.

**Writing – original draft:** Xianshuai Zhang, Kexin Yang, Siyi Wang.

**Writing – review & editing:** Zhenhua Li.
